# Genetic characterization of the first detected human case of avian influenza A (H5N6) in Anhui Province, East China

**DOI:** 10.1038/s41598-018-33356-4

**Published:** 2018-10-16

**Authors:** Jun He, Bo-Yu Liu, Lei Gong, Zhen Chen, Xiao-Long Chen, Sai Hou, Jun-Ling Yu, Jia-Bin Wu, Zhi-Cai Xia, Adams Latif, Rongbao Gao, Bin Su, Yan Liu

**Affiliations:** 1 Department of Microbiology, Anhui Medical University, Hefei, China; 2 Anhui Center for Disease Control and Prevention, Hefei, China; 3 Xuancheng City Center for Disease Control and Prevention, Xuancheng, China; 4 National Institute for Viral Disease Control and Prevention, Chinese Center for Disease Control and Prevention, Beijing, China

## Abstract

We compared complete genome sequences of two strains of an avian influenza A (H5N6) virus isolated from a patient in Anhui Province with those of other strains from GenBank and Global initiative on sharing all influenza data (GISAID). The HA gene of the isolated virus shared homology with that of A/chicken/Zhejiang/727155/2014 (H5N6) at the level of similarity of 98%. The six internal genes of the Anhui strains were close to those of H9N2 viruses from Zhejiang, Shandong, and Guangdong provinces, with a similarity of 99%. In addition, the similarity between the internal antigens (NP and MP) of the isolated H5N6 virus and H7N9 and H10N8 viruses was 99%. Based on the data of phylogenetic analysis, the H5N6 influenza virus isolated in Anhui Province belonged to clade 2.3.4.4. The virus was shown to have molecular characteristics of highly pathogenic avian influenza viruses, including eight glycosylation sites and an amino acid sequence of the HA protein cleavage site, PLRERRRKKR/GLF, containing multiple basic amino acids. Additionally, the stalk domain of the NA protein was found to have a deletion in NA stalk region (11 amino acids in N6, positions 58–68). Our study demonstrated that the H5N6 virus from Anhui Province represented a triple-reassortant virus and could be highly pathogenic to humans. The prevalence of this virus should be closely monitored.

## Introduction

In recent years, avian influenza viruses have been reported to frequently infect humans. Because of the high morbidity and mortality of the infection, avian influenza viruses have become a widespread public concern. Subtype H5N1, a highly pathogenic avian influenza (HPAI) virus, was first discovered in Guangdong Province, China, in 1996^1^, and the virus infected humans for the first time in 1997 in Hong Kong, China^2^. Currently, the haemagglutinin (HA) gene of the avian influenza A virus (H5 subtype) continues to evolve into multiple variants, and it reassorts with different neuraminidase (NA) genes to form many new subtypes. In 2008, a new reassortant virus – subtype H5N5 – was found in ducks^3^. After 2010, different subtypes of avian influenza A virus (i.e., H5N2, H5N6 and H5N8) were discovered in live poultry in different areas of China^4–6^. In April 2014, human infection with an avian influenza H5N6 virus was first reported in Sichuan Province of China^1^. In the same year, a second case was reported in Guangdong Province^7^. In the following year, two cases were reported in Yunnan Province in February and July of 2015^8^. In Anhui Province, the first human case of an avian influenza H5N6 strain was reported in Ningguo County, Xuancheng City in April 2016. To further understand the molecular characteristics and genetic relationship of these strains and to provide new information for disease prevention and control, we compared genetic characteristics of this latter case and those from Sichuan, Guangdong and Yunnan provinces.

## Materials and Methods

### Clinical and epidemiological data collection

A patient with severe pneumonia from Ningguo County, Xuancheng City, Anhui Province was suspected to be infected with a highly pathogenic avian influenza virus. Samples from the upper and lower respiratory tract were collected by clinicians at the Xuancheng Centre for Disease Control and Prevention in Anhui Province according to the biological safety requirements for cold chain transportation and carried to the Anhui Province Influenza Reference Center. Ethical approval for the study was granted by the human bioethics committee of the Anhui Provincial Center for Disease Control and Prevention and Anhui Medical University.

### Source and reference sequences

Two strains of H5N6 influenza virus, which were isolated from the upper and lower respiratory tract of the patient, were named A/Anhui/33162/2016 (H5N6) and A/Anhui/33163/2016 (H5N6), respectively. The complete genome sequences were obtained from the National Influenza Centre Sequence Database of China. The reference sequences were as follows: A/Sichuan/26221/2014 (H5N6) (SC26221), A/Guangzhou/39715/2014 (H5N6) (GZ39715), A/Changsha/1/2014 (H5N6) (CS1), and A/Yunnan/0127/2015 (H5N6) (YN0127). The GenBank or GISAID numbers of the above mentioned eight gene fragments were EPI533583-EPI533590, KP765785-KP765792, KR063684-KR063691 and KT245143-KT245150, respectively. The HA GenBank numbers of the A/Anhui/1/2005 (H5N1), A/Anhui/1/2006 (H5N1) and A/Anhui/1/2007 (H5N1) strains were DQ371928, CY098668 and CY098681, respectively.

### Sequence analysis

All sequences were trimmed using the BioEdit 7.0 software, and a phylogenetic tree was constructed by using the neighbour-joining method of the Molecular Evolutionary Genetics Analysis software (MEGA 6.0). A dendrogram was obtained based on genetic distances. All reference sequences were downloaded from NCBI or GISAID.

Amino acid sequence analysis was performed using the BioEdit 7.0 software. To identify the ORF region, DNA sequences were translated into protein sequences. The translated amino acid sequences were aligned using the MEGA 6.0 software, and amino acid sites were analysed.

## Results

### Epidemiological characteristics

The patient was a 65-year-old female. Due to fever, chill, cough and other symptoms, she went to the Jianmin Hospital of Ningguo County on April 25, 2016. Clinical examination showed that the patient had a fever (38 °C), slightly swollen throat, shortness of breath in both lungs, and right-lung auscultation. WBC was 8.6 × 10^9^ /L with 86% neutrophils. The patient was admitted to the hospital with unknown fever. The patient had a history of type II diabetes. She was treated with cefoxitin sodium and amikacin sulphate as anti-infection therapy, as well as with defervescence, phlegm-reducing and rehydration therapy for 2 days. Unfortunately, there was no obvious reduction of significant symptoms. On April 27, she was transferred to the Ningguo People’s Hospital for further diagnosis and treatment. On April 28, she was suspected to be infected with an avian influenza virus after expert consultation at the Wuhu Yijishan Hospital in Anhui Province. At the same time, her blood samples were sent to the Anhui Provincial Center for Disease Control and Prevention. On April 29, the provincial CDC confirmed that the patient was infected with subtype H5N6 of avian influenza virus A, and her samples were sent to the Chinese National Influenza Center for further confirmation. According to an epidemiological investigation, the patient was exposed to a dead chicken and a normal chicken before the onset of the disease, and she processed and ate the dead chicken.

### Analysis of similarity of gene fragments

We cultured the H5N6 virus from the upper and lower respiratory tract samples of the patient and isolated two strains of the virus (AH33162 and AH33163). Using Sanger sequencing on an ABI 3730XL analyser, we sequenced eight genomic fragments of each isolate and found 100% homology between the nucleotide sequences of the two strains. Strains with the highest sequence similarity, based on NCBI BLAST, are shown in Table 1. The HA gene of our viral strains showed high homology with that of A/chicken/Zhejiang/727155/2014 (H5N6) virus, with 98% similarity. The sequences of the six internal genes were close to those of H9N2 viruses from Zhejiang, Shandong, and Guangdong, with 99% similarity. Additionally, the sequence similarity of the internal antigens (NP and MP) with those of H7N9 and H10N8 strains was 99%.

**Table 1:** Similarity analysis of the H5N6 virus sequence from the first avian influenza case in Anhui Province.

Gene	Length (bp)	Strain with highest similarity	GenBank ID	Similarity (%)
PB2	2280	A/chicken/Zhejiang/SIC40/2015 (H9N2)	KX598552	99
PB1	2274	A/chicken/Zhejiang/SIC40/2015 (H9N2)	KX598594	99
PA	2151	A/chicken/Zhejiang/SIC30/2014 (H9N2)	KX598621.	99
HA	1776	A/chicken/Zhejiang/727155/2014 (H5N6)	KU042766	98
NP	1561	A/chicken/Wenzhou/HATSLG01/2015 (H7N9)	KU143421	99
		A/chicken/Qingdao/018/2014 (H9N2)	KT449649	99
NA	1380	A/chicken/Zhejiang/727022/2014 (H5N6)	KU042822	98
MP	982	A/chicken/Jiangxi/18513/2014 (H7N9)	KP418439	99
		A/chicken/Dongguan/1674/2014 (H9N2)	KP416449	99
		A/chicken/Hebei/SJZ01/2015 (H10N8)	KY402022	99
NS	861	A/Anser fabalis/Anhui/L139/2014 (H9N2)	KT699060	99

### Analysis of genetic evolution of gene fragments

**Figure 1:**
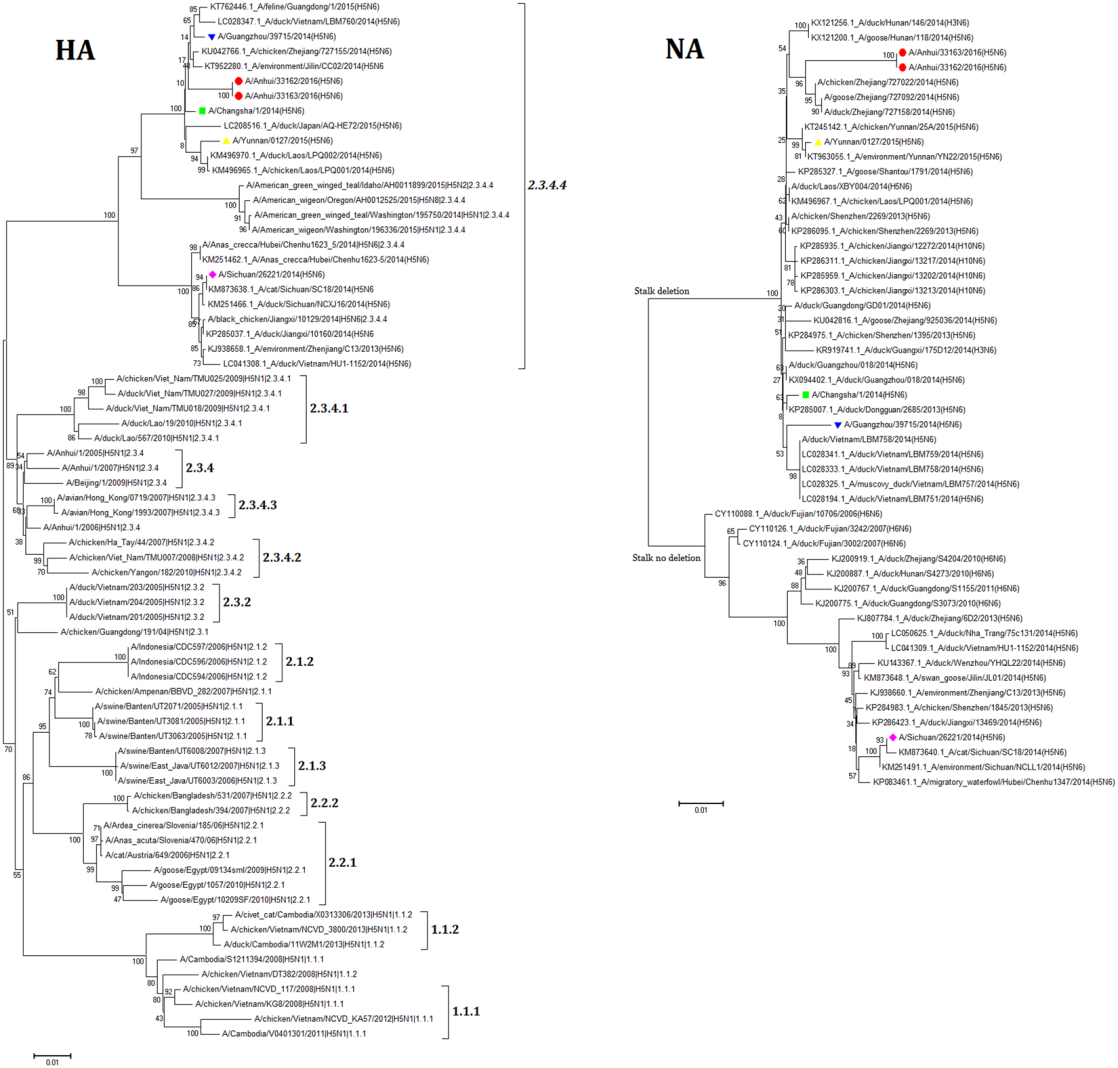
Phylogenetic trees of avian influenza A viruses, based on the haemagglutinin (HA) and neuraminidase (NA) genes. Neighbour-joining trees were constructed using the Molecular Evolutionary Genetics Analysis software (MEGA 6.0). A dendrogram was obtained based on genetic distances. All reference sequences were downloaded from NCBI, GISAID or the National Influenza Centre of China. Bootstrap values were calculated for 1,000 replicates. A/Anhui/33162/2016(H5N6) and A/Anhui/33163/2016(H5N6) were indicated with red dots. A/Sichuan/26221/2014(H5N6), A/Yunnan/0127/2015(H5N6), A/Guangdong/39715/2014(H5N6) and A/Changsha/1/2014(H5N6) were indicated with a pink rhombus, a yellow positive trigonometric, a blue inverted triangle and a green square, respectively. Brackets denote H5 subtype virus clades. Scale bars indicate nucleotide substitutions per site.

A phylogenetic tree based on genetic evolution of the avian influenza virus (H5N6) HA gene showed that the H5N6 influenza virus from Anhui Province belonged to clade 2.3.4.4 (Fig. 1), which is the same clade that includes strains from Sichuan, Changsha, Guangzhou and Yunnan provinces. Clade 2.3.4.4 comprises three different sub-clades, among which the human influenza H5N6 virus from Sichuan Province forms a separate sub-clade, while the H5N6 viruses from Anhui, Guangzhou, Yunnan and Changsha were found in the same sub-clade, which is separated from that formed by avian influenza viruses from birds and the environment from Guangdong, Zhejiang, and Jilin provinces of China, as well as from Japan, Laos, and Vietnam. The avian influenza virus (H5N6) isolated from American waterfowl is in another sub-clade. It is noteworthy that clade 2.3.4.4 includes not only subtype H5N6 of avian influenza virus A but also subtypes H5N1, H5N2, and H5N8 (Fig. 1). An analysis of genetic evolutionary relationships of NA revealed that the H5N6 cases from Anhui, Guangzhou, Yunnan, and Changsha were in a different clade from the Sichuan cases. In the Anhui clade, there are subtypes H5N6, H3N6 and H10N6 of avian influenza virus A, while subtypes H5N6 and H6N6 are in the Sichuan clade (Fig. 1).

### Analysis of key amino acids

We compared three strains of human influenza virus (H5N1) isolated during the years of 2005~2007 in Anhui Province with the four H5N6 strains from Sichuan, Guangzhou, Changsha and Yunnan. The results of comparative analysis are shown in Tables 2 and 3. Based on the evolutionary relationships of the HA (H5N1) protein, the three human strains belonged to clade 2.3.4, and the four H5N6 strains belonged to clade 2.3.4.4. Compared with H5N1 from clade 2.3.4, H5N6 has six amino acid substitutions in the receptor-binding site (RBS), which are the same in viruses from clade 2.3.4.4. There are two substitutions in the 130-loop (S133L and S137A), three substitutions located in the 190-helix (D187N, K193N and Q196K), and one in the 220-loop (S227R). The H5N6 virus from Anhui Province has eight glycosylation sites, while other H5N6 and H5N1 viruses have seven glycosylation sites in our reference strains. However, the H5N6 virus from Sichuan Province has only six glycosylation sites. Because of the D129N or E131T substitutions in the HA protein, the H5N6 viruses from Anhui, Changsha and Yunnan acquired one glycosylation site, at 129, comparing with the isolated viruses of H5N1 from Anhui in 2005, 2006 and 2007, but the T160A mutation resulted in a loss of one glycosylation site in the H5N6 viruses from Sichuan, Changsha and Yunnan, at 158.

**Table 2:** Molecular features of the HA gene in H5N6 viruses isolated from avian influenza cases.

Strain name	Receptor-binding site							Cleavage site	Glycosylation site							
	133	137	160	187	193	196	226–228		21	33	129	158	169	289	483	541
AH33162	Del	A	S	N	K	K	QSG	PLRERRRKKR/GLF	NST	NVT	NHT	NDS	NNT	NSS	NGT	NGS
AH33163	Del	A	S	N	K	K	QSG	PLRERRRKKR/GLF	NST	NVT	NHT	NDS	NNT	NSS	NGT	NGS
SC26221	L	A	A	N	K	K	QRG	PLREKRRKR/GLF	NST	NVT	—	—	NNT	NSS	NGT	NGS
GZ39715	L	A	T	N	K	K	QRG	PLRERRRKR/GLF	NST	NVT	—	NDT	NNT	NSS	NGT	NGS
CS1	Del	A	A	N	K	K	QRG	PLRERRRKR/GLF	NST	NVT	NHT	—	NNT	NSS	NGT	NGS
YN0127	Del	A	A	N	K	K	QRG	PLRERRRKR/GLF	NST	NVT	NHT	—	NNT	NSS	NGT	NGS
AH1/2005	S	S	T	D	N	Q	QSG	PLRERRRKR/GLF	NST	NVT	—	NNT	NNT	NSS	NGT	NGS
AH1/2006	S	S	T	D	N	Q	QSG	PLRERRRKR/GLF	NST	NVT	—	NNT	NNT	NSS	NGT	NGS
AH1/2007	S	S	T	D	N	Q	QSG	PLRERRRKR/GLF	NST	NVT	—	NNT	NNT	NSS	NGT	NGS

Abbreviations: SC26221, A/Sichuan/26221/2014 (H5N6); GZ39715, A/Guangzhou/39715/2014 (H5N6); CS1, A/Changsha/1/2014 (H5N6); YN0127, A/Yunnan/0127/2015 (H5N6); AH1/2005, A/Anhui/1/2005 (H5N1); AH1/2006, A/Anhui/1/2006 (H5N1); AH1/2007, A/Anhui/1/2007 (H5N1); AH33162, A/Anhui/33162/2016 (H5N6); AH33163, A/Anhui/33163/2016 (H5N6); -, None.

**Table 3:** Molecular features of the other genes of H5N6 viruses isolated from avian influenza cases.

Protein	Phenotypic effect	Site substitution or molecular feature	AH33162	AH33163	SC26221	GZ39715	CS1	YN0127	Reference
NA (N6 numbering)	Increased virulence in mice	Stalk deletion	Yes	Yes	No	Yes	Yes	Yes	^14, 15^
PB2	Enhanced polymerase activity and increased virulence	E627K	K	K	E	K	E	K	^16, 22^
		D701N	D	D	N	D	D	D	
		L89V	V	V	V	V	V	V	
PB1	Associated with H5 transmissibility	I368V	V	V	I	I	I	V	^16, 17^
PA	Adaptation to a mammalian host	A343T	A	A	A	A	A	A	^23^
M1	Increased virulence in mice	N30D	D	D	D	D	D	D	^18, 19^
		T215A	A	A	A	A	A	A	
M2	Anti-viral amantadine resistance	S31N	N	N	S	S	N	N	^24^
NS1	Increased virulence in mice	P42S	S	S	S	S	S	S	^18, 19^
		80~84 deletion	NO	NO	Yes	Yes	Yes	NO	^25^

The amino acid sequence of the HA protein cleavage site of the Anhui H5N6 influenza virus is PLRERRRKKR/GLF, thus containing multiple basic amino acids and indicating that the virus possesses molecular characteristics of highly pathogenic avian influenza viruses. The stalk domain of the NA protein has a deletion in NA stalk region (11 amino acids in N6, positions 58–68). The H5N6 viruses from Guangzhou, Yunnan and Changsha have the same deletion at this position. However, the virus from Sichuan has no deletion, and the H274Y and R292K viral substitutions were not found, which suggests that the virus retains the sensitivity to neuraminidase inhibitors. However, the S31N substitution was detected in the M2 protein, suggesting the occurrence of drug resistance to amantadine. Additionally, E627K and L89V substitutions were found in the PB2 protein, normally seen in mammal-adapted AIVs, but no D701N substitution was detected. The I368V substitution occurred in the PB1 protein, associated with H5 transmissibility, but there was no H99Y substitution. We also found that N30D and T215A substitutions occurred in the M1 protein, and the P42S substitution occurred in the NS1 protein, which were related to the virulence increase of the virus in mice, but no deletion of 80~84 amino acids was found (Table 3).

## Discussion

Based on the genetic evolutionary relationships, the H5N6 influenza virus from Anhui Province represents a triple-reassortant virus. Its HA was derived from an H5 subtype avian influenza virus from clade 2.3.4.4, and NA was derived from subtype H5N6, H3N6 or H10N6. The six internal genes of the virus were derived from an H9N2 avian influenza virus. NAs of the H5N6 strains from Anhui, Guangzhou, Changsha and Yunnan, containing a deletion in NA stalk region (11 amino acids in N6, positions 58–68), are clearly in a different clade from that of NA of the Sichuan strain, with no deletion at the same sites, suggesting that there are two groups of H5N6 viruses, with different gene reassortment, which continue to evolve. It is difficult to speculate which H5N6 genotype will form the dominant strain.

In recent years, avian influenza viruses of subtype H5 from clade 2.3.4.4 have been very active. In addition to H5N6, there are subtypes H5N1, H5N2 and H5N8 forming a large gene pool, which facilitates the selection of different viral NAs for gene reassortment. During 2014~2015, there were fourteen reported cases of infection by these avian influenza viruses, which causes a widespread concern^9^. H5N6 viruses from clade 2.3.4.4 show a broad geographical distribution. Not only have they been found in many provinces in China, but the viruses were also isolated in neighbouring countries such as Laos, Vietnam and Japan. Recent studies have found that H5N6 viruses can infect wild birds and domestic cats^10^, suggesting that the bird migration may be an important factor causing the widespread prevalence of subtype H5, while the detection of infection of domestic cats implies that the virus may adapt to mammalian hosts upon effective multiplication. H9N2 viruses have provided six internal genes for H5N1, H7N9, and H10N8 viruses^11^. The six internal genes of the H5N6 virus isolated from the case in Anhui Province are highly homologous to those of subtype H9N2. Because of the current widespread occurrence of low pathogenic avian influenza viruses (H9N2), they are likely to form new virus subtypes with highly pathogenic avian influenza viruses, and there is a risk of an influenza pandemic.

Characterization of viral genes revealed six mutations that occurred in the receptor-binding site of the HA protein of the H5N6 virus from Anhui Province, in contrast with that of the H5N1 virus from clade 2.3.4; however, we did not find deglycosylation in our isolated strains, which enhances mammalian receptor affinity, at the 158 site^12^. In addition, we found no Q226L substitution, which is associated with mammalian receptor affinity^13^. The stalk domain deletion in the NA protein is associated with the enhancement of the viral replication ability and pathogenicity to mice, as well as with a better ability to be transmitted in poultry^14,15^. The PB2 protein with the E627K and L89V substitutions and PB1 with the I368V substitution are associated with increased adaptation to mammalian transmission and with viral replication^16,17^. The M1 protein with the N30D and T215A substitutions and NS1 with the P42S substitution can increase the viral virulence in mice^18,19^. We speculated that the isolated virus only acquired a partial ability to adapt to mammals but had molecular characteristics allowing it to widely spread in poultry. Meanwhile, the virus has many enhanced pathogenic mutations, and thus, its prevalence and pathogenic characteristics in animals should be closely monitored.

Outbreaks of human infections caused by new avian influenza viruses have continued in recent years, and for some viruses (H5N1 and H7N9), limited human-to-human transmission has been observed^20,21^. So far, there have been no reports of human-to-human transmission for subtype H5N6 of avian influenza viruses. It should be noted that this virus has been able to spread in live poultry, which plays an important role in the process of evolution of avian influenza viruses. The virus is likely to undergo further geographic spread in the future. Therefore, controlling the market of wild birds and live poultry and analysing virus evolution and gene mutations in a timely manner are essential.
